# Supplementary material for: Universal loop assembly: open, efficient and cross-kingdom DNA fabrication
Source: Synth Biol (Oxf). 2020 Feb 5;5(1):ysaa001. doi: 10.1093/synbio/ysaa001 (PMC7052795; doi:10.1093/synbio/ysaa001)
Supplement: ysaa001_Supplementary_Data [file ysaa001_supplementary_data.pdf]

Supplementary Data

"Universal Loop assembly (uLoop): open, efficient, and species-agnostic DNA fabrication"

Bernardo Pollak*, Tamara Matute, Isaac Nunez, Ariel Cerda, Constanza Lopez, Valentina Vargas, Anton Kan, Vincent Bielinski, Peter von Dassow, Chris L. Dupont and Fernan Federici*.

* Correspondence to bpollakw@gmail.com and ffederici@bio.puc.cl.

Supplementary Figures

Supplementary Figure S1. Diagram of uLoop assembly logic. 1
Supplementary Figure S2. Vector backbone refactoring. 2
Supplementary Figure S3. Representative plate images used for analysis of assembly efficiency and productivity. 2
Supplementary Figure S4. Integrity of assembly. 3
Supplementary Figure S5. Reaction setup tests. 4
Supplementary Figure S6. Stability of expression of uLoop plasmid in *P.tricornutum*. 5
Supplementary Figure S7. Histograms of *P.tricornutum* ex-conjugate fluorescence before and after cell sorting. 6
Supplementary Figure S8. Use of uLoop vectors across multiple organisms. 7
Supplementary Figure S9. AKR1 green bleed-trough estimation. 14
Supplementary Figure S10. Red fluorescence bleed-trough estimation. 15
Supplementary Figure S11. GFP background subtraction. 15
Supplementary Figure S12. RFP background subtraction. 16

Supplementary Tables

Supplementary Table S1. Green-to-red fluorescent ratio values for *E.coli* plate fluorometry. 8
Supplementary Table S2. ANOVA test for *E.coli* plate fluorometry Green/Red fluorescent ratio values between levels across the four kits (pCA, pSB, pCO, pAN). 8
Supplementary Table S3. ANOVA test for E. coli plate fluorometry Green/Red fluorescent ratio values comparing levels within each plasmid kit. 9
Supplementary Table S4. pCA multiple comparison of means (Tukey HSD test) for E. coli plate fluorometry Green/Red fluorescent ratio values. 9
Supplementary Table S5. Internal group ANOVA test for *E. coli* flow cytometry Green/Red fluorescent ratio values. 9
Supplementary Table S6. pAN and pCA multiple comparison of means (Tukey HSD test) for *E.coli* flow cytometry Green/Red fluorescent ratio values. 9
Supplementary Table S7. Green-to-red fluorescent ratio values for *E.coli* plate fluorometry and flow cytometry by plasmid group. 10
Supplementary Table S8. *E. coli* growth rate values. 10
Supplementary Table S9. ANOVA test for E. coli plate fluorometry growth rate values. 10
Supplementary Table S10. L0 part list. 11
Supplementary Table S11. L1 assembly list. 12
Supplementary Table S12. L2, L3 and L4 assembly list 13

Supplementary Text

Plate fluorometry data analysis 14
Growth rate measurements 17

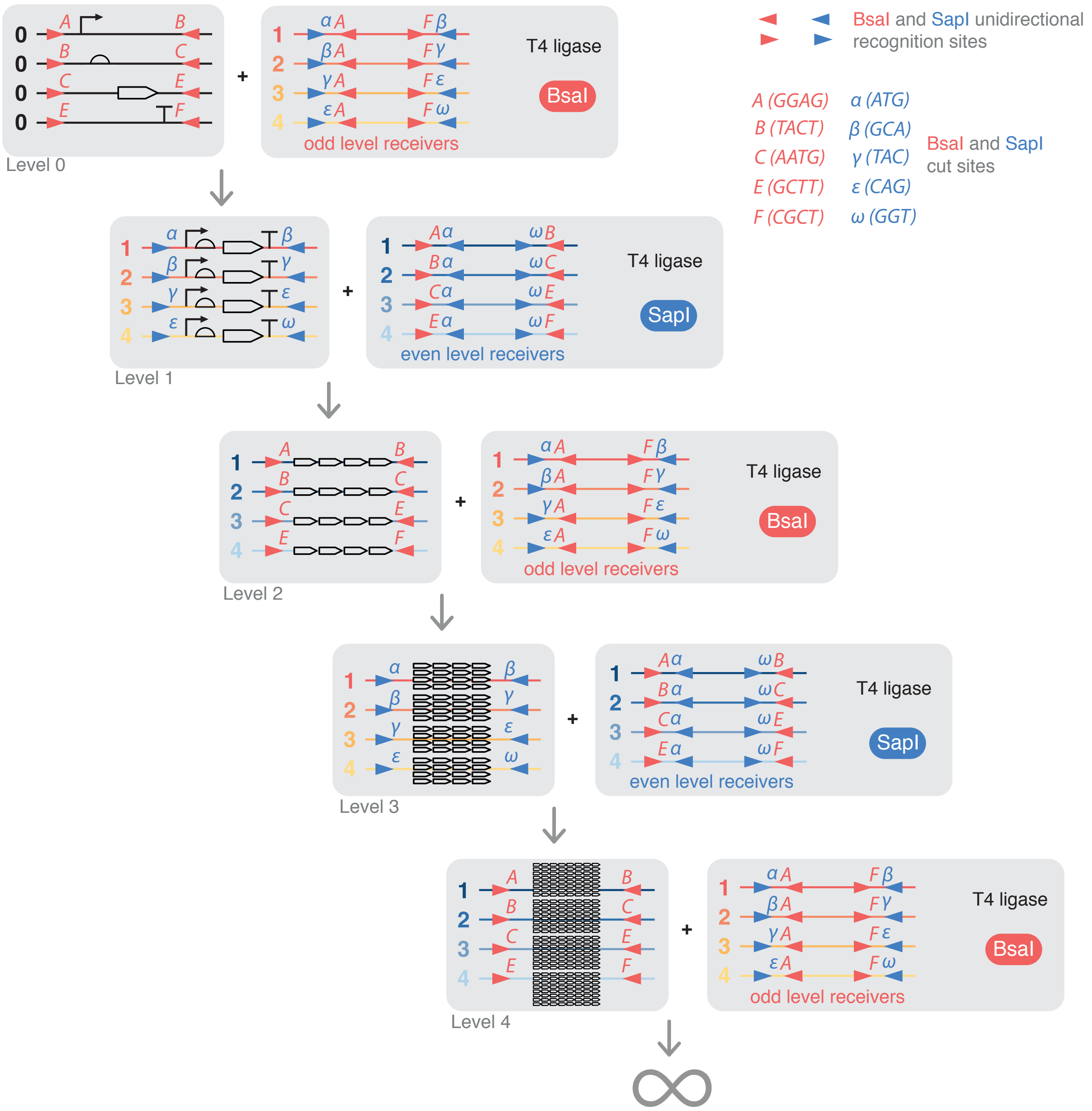

Supplementary Figure S1. Diagram of uLoop assembly logic.

uLoop is composed of two sets of four plasmids, called even and odd receivers (shown in red and blue, respectively). Vectors of exponentially increasing size are built by alternating BsaI and SapI Golden Gate assembly reactions through the repeated use of odd and even vectors. The use of inverted orientations for BsaI and SapI recognition sites in odd and even receivers permits the product of one reaction to become the substrate for the next reaction at the following level. Up to four assemblies can be conducted in parallel per level, allowing DNA elements to be composed in an exponential manner of 4 genetic modules per assembly round. BsaI overhangs follow the common syntax [1, 2], and SapI overhangs are the same as those described in Loop assembly [3], $\alpha(ATG), \beta(GCA), \gamma(TAC), \alpha(CAG)$ and $\omega(GGT)$ for hierarchical and sequential assembly in repetitive loops.

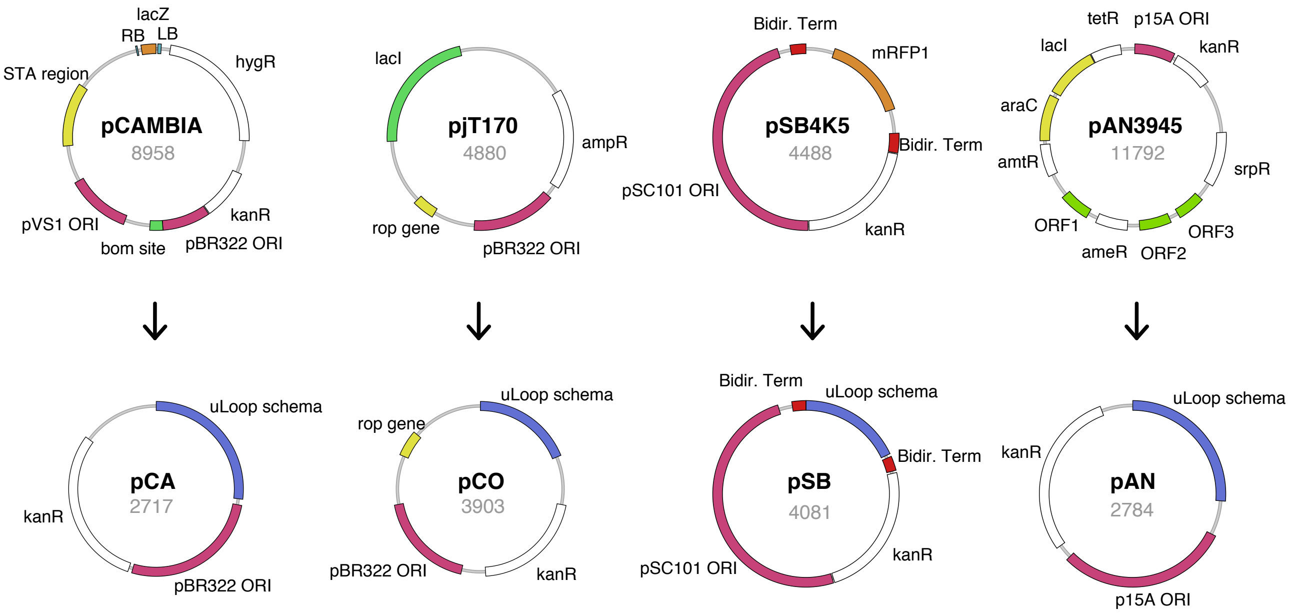

Supplementary Figure S2. Vector backbone refactoring.
Vectors from the synthetic biology community (pjT170, pSB4K5 and pAN3945) and the pCAMBIA vector were domesticated for BsaI and SapI and 'minimised' by removing elements not related to basic plasmid function. Numbers below the vector name denote the size in base pairs.

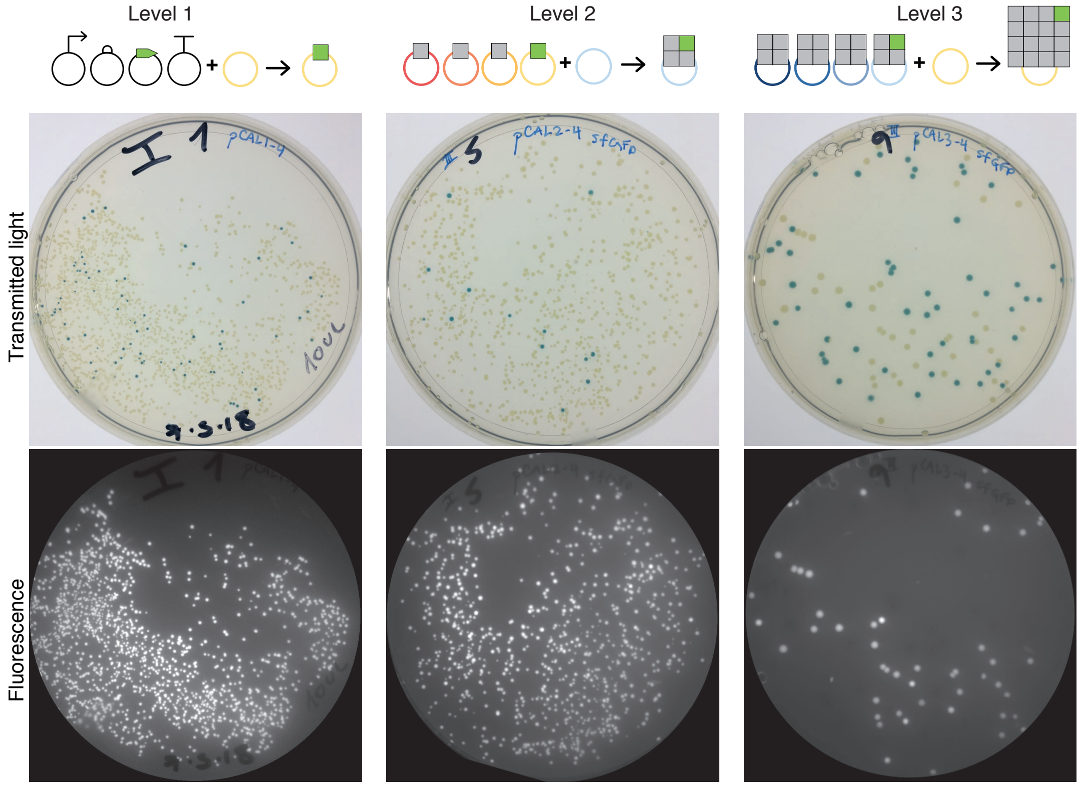

Supplementary Figure S3. Representative plate images used for analysis of assembly efficiency and productivity.
Resulting plates for L1 (left), L2 (middle) and L3 (right) assembly reactions in pCA vectors are shown. Transmitted light images show the presence of blue colonies that correspond to the negative background of the reactions given by the presence of the negative selection marker LacZ (top panels). In each assembly a single bacterial sfGFP expression cassette enables visualisation of fluorescent colonies in plates under UV illumination (bottom panels).

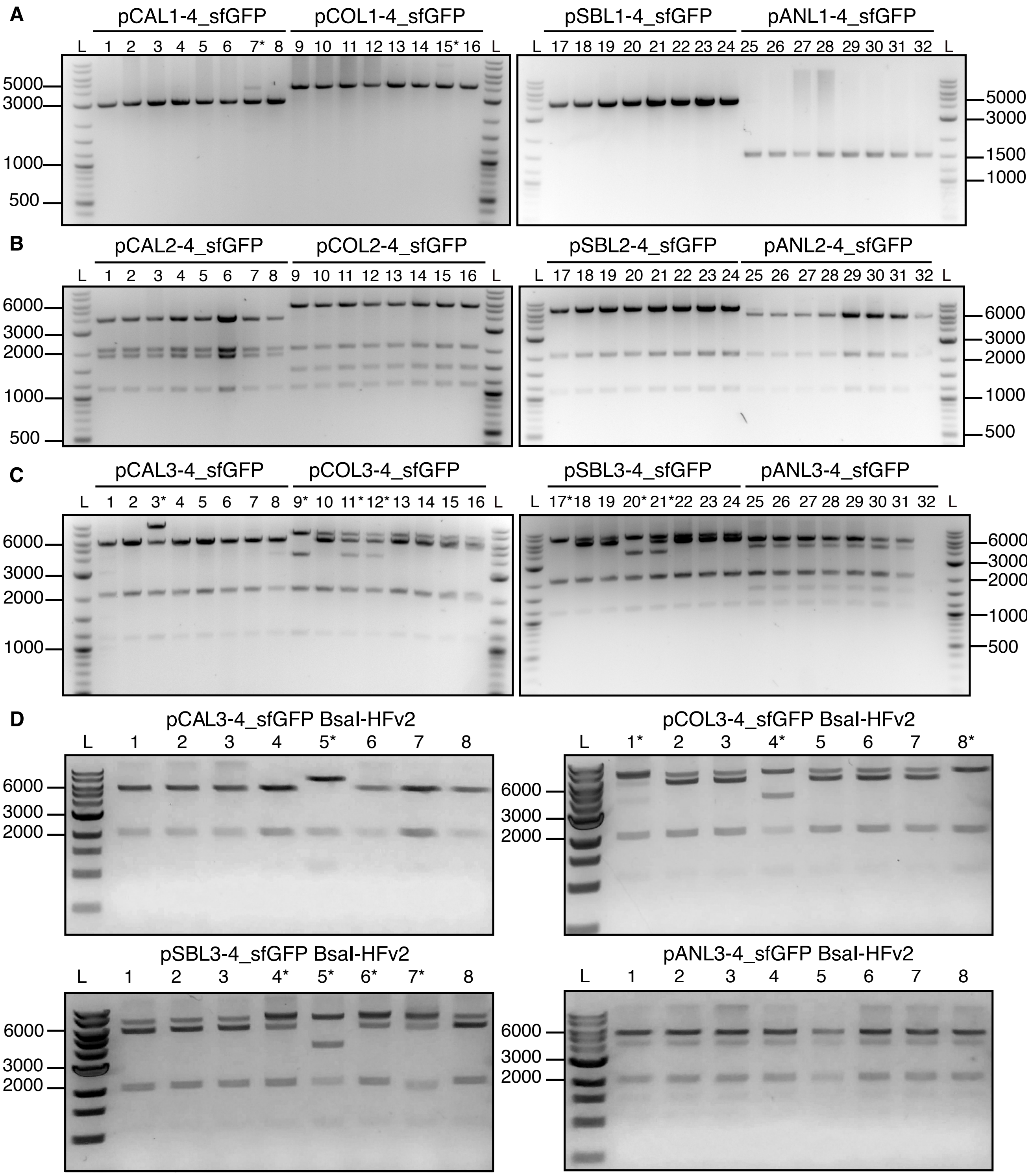

Supplementary Figure S4. Integrity of assembly.

Purified assemblies were evaluated by restriction profiling to determine integrity of assembly by comparison with expected digestion profiles. Incorrect assemblies are designated with an * next to the sample number. (A) Integrity of L1 assemblies. Purified L1 assemblies were digested with HindIII and restriction profiles determined by agarose-gel electrophoresis. (B) Integrity of L2 assemblies. Purified L2 assemblies were digested with XbaI and restriction profiles determined by agarose-gel electrophoresis. (C) Integrity of L3 assemblies. Purified L3 assemblies were digested with HindIII and restriction profiles determined by agarose-gel electrophoresis. (D) Integrity of L3 assemblies using BsaI-HFv2. Purified L3 assemblies generated with BsaI-HFv2 were digested with HindIII and restriction profiles determined by agarose-gel electrophoresis.

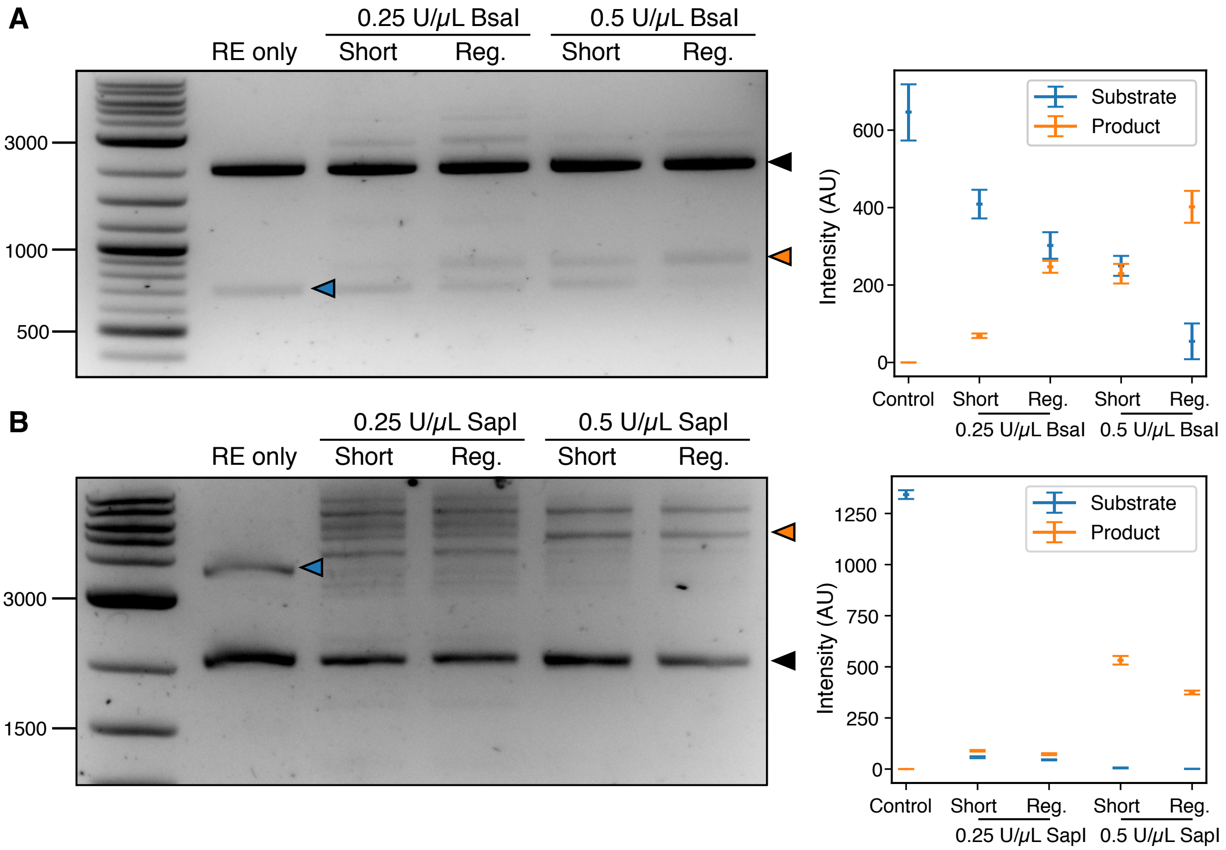

Supplementary Figure S5. Reaction setup tests.

(A) BsaI tests. An image for a resulting agarose gel electrophoresis of BsaI concentration and cycling condition tests is shown along with a lineplot with the quantification of the expected substrate and product bands of the reaction. The black arrow indicates the cut vector backbone, the blue arrow indicates the substrate and the orange arrow indicates the expected full-length product. (B) SapI tests. An image for a resulting agarose gel electrophoresis of BsaI concentration and cycling condition tests is shown along with a lineplot with the quantification of the expected substrate and product bands of the reaction. The black arrow indicates the cut vector backbone, the blue arrow indicates the substrate and the orange arrow indicates the expected full-length product.

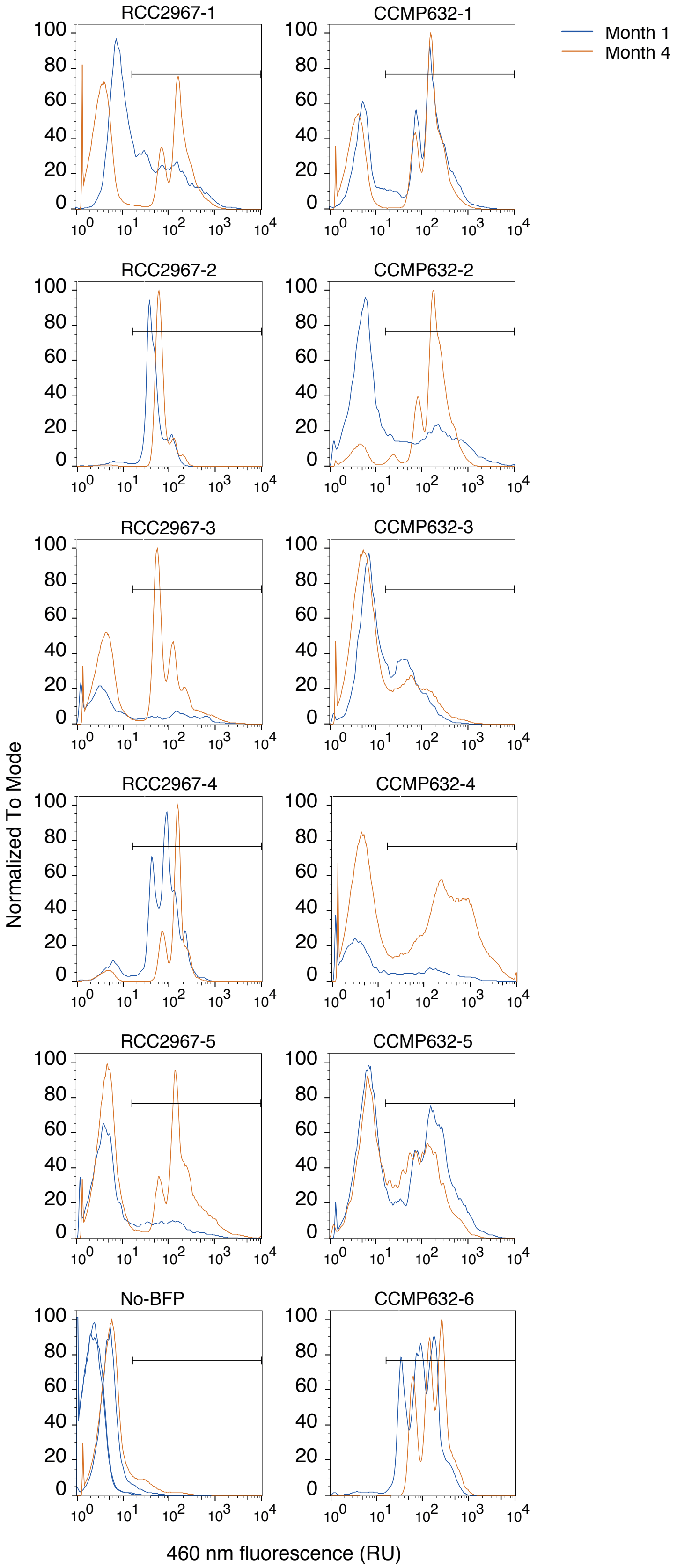

Supplementary Figure S6. Stability of expression of uLoop plasmid in *P.tricornutum*. Histograms of blue fluorescence (exc. 405 nm, em. 460/30 nm) of ex-conjugates of *P. tricornutum* sub-strains RCC2967 and CCMP632 expressing mTagBFP2 under the control of the H4 promoter, tested after first growth in liquid medium (month 1) and three months later (month 4). Bar shows the gate defining blue fluorescent cells, with the lower limit based on the maximum 95th percentile of blue fluorescence in no-BFP controls (exconjugates with a plasmid containing the nonfluorescent luxR gene) run in parallel with the same cytometer settings.

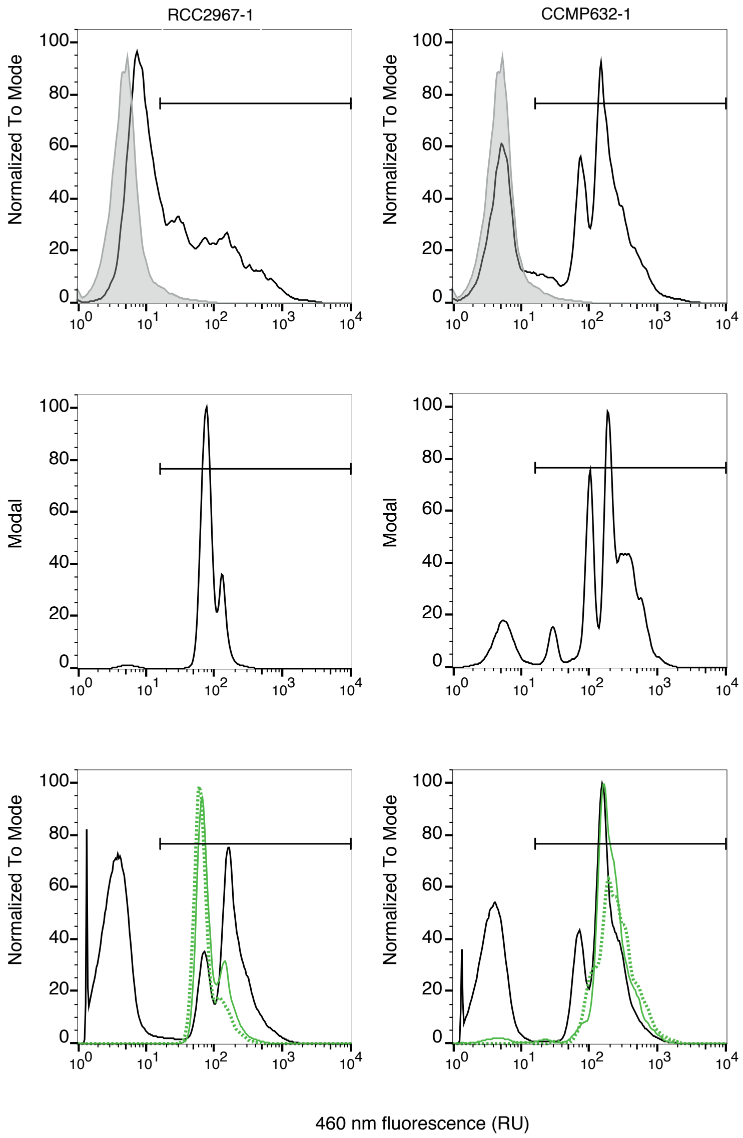

Supplementary Figure S7. Histograms of *P.tricornutum* ex-conjugate fluorescence before and after cell sorting. Top two panels: Histograms at month 0, without sorting. Also shown for comparison is the histogram of ex-conjugates with the gene for the non-fluorescent protein LuxR (light grey). Middle two panels: 2.5 months after conjugation, when blue-fluorescent cells were sorted. Bottom two panels: 4 months after conjugation, comparing cultures from sorted cells (green) to original ex-conjugates (black), both allowed to evolve through several transfers in liquid medium with zeocin. Bar shows the gate defining blue fluorescent cells, with the lower limit based on the maximum 95th percentile of blue fluorescence in No-BFP controls (exconjugates with a plasmid containing the nonfluorescent luxR gene) run in parallel with the same cytometer settings.

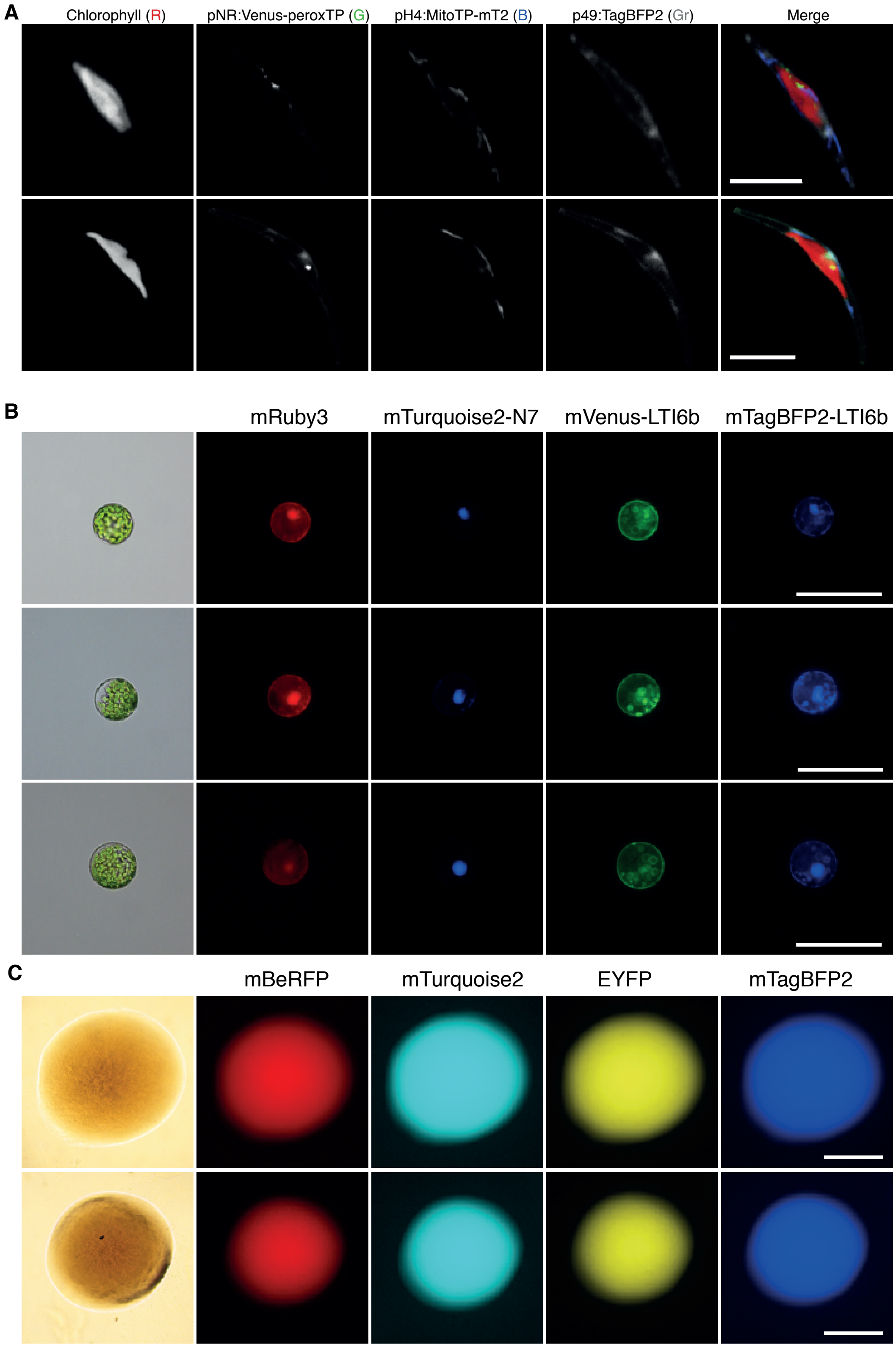

Supplementary Figure S8. Use of uLoop vectors across multiple organisms.
(A) Two representative individuals of *P. tricornutum* exconjugants transformed with pCAL2-1-FPrep showing expression of three fluorescent reporters. From left to right, Chlorophyll fluorescence, mVenus fluorescent protein fused to a peroxisomal localisation tag, mTurquoise2 fluorescent protein fused to a mitochondrial localisation tag and mTagBFP2 fluorescent protein expressed in the cytoplasm. Scale bar = 10 μm. (B) Three representative protoplasts of *Arabidopsis thaliana* transformed with pSBL2-1-4xFP vector harboring four fluorescent reporters. From left to right, mRuby3 fluorescent protein expressed in the cytoplasm, mTurquoise2 fluorescent protein fused to the nuclear localisation tab N7, mVenus fluorescent protein fused to plasma-membrane localisation signal LTi6b and mTagBFP2 fluorescent protein fused to plasma-membrane localisation signal LTi6b. Scale bar = 100 μm. (C) Expression of four fluorescent reporters in *Escherichia coli* colonies transformed with showing expression of mBeRFP, mTurquoise2, EYFP and mTagBFP2 fluorescent proteins pCA (top) and pSB (bottom) uLoop vectors. Scale bar = 500 μm.

Plasmid	Level	Mean	STD	SEM	95% CI
pCA	Level 1	4,43	0,53	0,31	[3,12 - 5,75]
pCA	Level 2	5,81	1,01	0,58	[3,29 - 8,32]
pCA	Level 3	2,57	0,42	0,25	[1,51 - 3,62]
pCO	Level 1	2,28	0,50	0,29	[1,04 - 3,52]
pCO	Level 2	2,80	0,54	0,31	[1,46 - 4,13]
pCO	Level 3	2,05	0,44	0,25	[0,97 - 3,13]
pSB	Level 1	5,02	0,75	0,43	[3,15 - 6,88]
pSB	Level 2	4,33	0,65	0,37	[2,72 - 5,94]
pSB	Level 3	4,18	0,49	0,28	[2,97 - 5,39]
pAN	Level 1	0,03	0,03	0,02	[-0,04 - 0,10]
pAN	Level 2	0,41	0,31	0,18	[-0,37 - 1,18]
pAN	Level 3	0,23	0,20	0,12	[-0,27 - 0,73]
AKR1	-	0,00	0,00	0,00	[0,00 - 0,00]

Supplementary Table S1. Green-to-red fluorescent ratio values for *E.coli* plate fluorometry.
The mean, standard deviation (STD), standard error of the mean (SEM) and 95% confidence intervals are shown for each plasmid level and group. AKR1 does not contain 'Level' plasmids, thus only the group value is reported. Values correspond to three independent replicates (N=3).

	DF	SS	MSS	F	PR(>F)
**Level**	2.0	7.141522	3.570761	0.941299	0.400345
**Residual**	33.0	125.183476	3.793439		

Supplementary Table S2. ANOVA test for *E.coli* plate fluorometry Green/Red fluorescent ratio values between levels across the four kits (pCA, pSB, pCO, pAN).
A two-way ANOVA comparison was performed for L1, L2 and L3 values of the four kits (pCA, pSB, pCO, pAN) grouped together per level. There were no statistically significant differences between group means (i.e. plasmid sizes). DF: degrees of freedom; SS: sum of squares; MSS: mean sum of squares; F: F statistic value; PR(>F): p-value of F statistic. AKR1 data was not considered.

Plasmid set		DF	SS	MSS	F	PR(>F)
**pCA**	**Level**	2.0	15.866.420	793.321	16.008.907	0.003931
	**Residual**	6.0	2.973.299	0.49555		
**pCO**	**Level**	2.0	0.876727	0.438363	1.803.301	0.243638
	**Residual**	6.0	1.458.537	0.243089		
**pSB**	**Level**	2.0	1.187.523	0.593761	1.461.676	0.303997
	**Residual**	6.0	2.437.317	0.406220		
**pAN**	**Level**	2.0	0.213126	0.106563	2.301.536	0.1812
	**Residual**	6.0	0.277804	0.046301		

Supplementary Table S3. ANOVA test for E. coli plate fluorometry Green/Red fluorescent ratio values comparing levels within each plasmid kit.
A two-way ANOVA comparison was performed among L1, L2 and L3 values for each plasmid kit. There were statistically significant differences between levels of pCA kit. DF: degrees of freedom; SS: sum of squares; MSS: mean sum of squares; F: F statistic value; PR(>F): p-value of F statistics.

Group 1	Group 2	Mean Diff	Lower	Upper	p-value	Reject
Level 1	Level 2	1.37	-0.39	3.13	0.1174	False
Level 1	Level 3	-1.87	-3.63	-0.11	0.0399	True
Level 2	Level 3	-3.24	-5.00	-1.48	0.0032	True

Supplementary Table S4. pCA multiple comparison of means (Tukey HSD test) for E. coli plate fluorometry Green/Red fluorescent ratio values.
Using rejection under 95% significance for each pair compared, showed statistically significant differences for level 3 of pCA.

Plasmid set		DF	SS	MSS	F	PR(>F)
pCA	Level	2	2.401	1.200	11.491	0.009
	Residual	6	0.627	0.104		
pCO	Level	2	0.149	0.074	1.115	0.387
	Residual	6	0.400	0.067		
pSB	Level	2	0.4756	0.2378	2.6964	0.1461
	Residual	6	0.5292	0.0882		
pAN	Level	2	0.3474	0.1737	12.6725	0.0070
	Residual	6	0.0822	0.0137		

Supplementary Table S5. Internal group ANOVA test for *E. coli* flow cytometry Green/Red fluorescent ratio values.
A two-way ANOVA comparison was performed among L1, L2 and L3 values for each plasmid kit. There were statistically significant differences between uLoop levels of pCA and pAN vector kits. DF: degrees of freedom; SS: sum of squares; MSS: mean sum of squares; F: F statistic value; PR(>F): p-value of F statistic.

Vector Set	Group 1	Group 2	Mean Diff	Lower	Upper	p-value	Reject
**pAN**	pANL1	pANL2	0,401	0,108	0,694	0,0135	**True**
	pANL1	pANL3	-0,030	-0,323	0,263	0,9000	False
	pANL2	pANL3	-0,431	-0,724	-0,138	0,0096	**True**
**pCA**	pCAL1	pCAL2	0,239	-0,570	1,047	0,6521	False
	pCAL1	pCAL3	-0,957	-1,766	-0,148	0,0255	**True**
	pCAL2	pCAL3	-1,195	-2,004	-0,387	0,0094	**True**

Supplementary Table S6. pAN and pCA multiple comparison of means (Tukey HSD test) for *E.coli* flow cytometry Green/Red fluorescent ratio values.
Rejection was performed under 95% significance for each pair compared.

		Plate fluorometry			Flow cytometry		
**Plasmid**	**Level**	**Mean**	**STD**	**CV**	**Mean**	**STD**	**CV**
pCA	All	4,27	1,53	35,95	1,50	0,62	40,99
pCO	All	2,37	0,54	22,75	1,37	0,26	19,16
pSB	All	4,51	0,67	14,93	1,67	0,35	21,28
pAN	All	0,22	0,25	111,31	0,57	0,23	40,55

Supplementary Table S7. Green-to-red fluorescent ratio values for *E.coli* plate fluorometry and flow cytometry by plasmid group.
The mean, standard deviation (STD), and coefficient of variation (CV) are shown for each vector group. Values correspond to three independent replicates and three levels per group (N=9).

**Plasmid**	**Level**	**Mean**	**STD**	**SEM**	**95% CI**
pCA	Level 1	1,26	0,20	0,12	[0,76 - 1,76]
pCA	Level 2	1,25	0,14	0,08	[0,89 - 1,60]
pCA	Level 3	1,33	0,15	0,09	[0,94 - 1,71]
pCA	All	1,28	0,15	0,05	[1,16 - 1,39]
pCO	Level 1	1,35	0,05	0,03	[1,23 - 1,46]
pCO	Level 2	1,34	0,19	0,11	[0,86 - 1,82]
pCO	Level 3	1,42	0,17	0,10	[1,01 - 1,83]
pCO	All	1,37	0,14	0,05	[1,26 - 1,47]
pSB	Level 1	1,17	0,14	0,08	[0,81 - 1,52]
pSB	Level 2	1,32	0,20	0,12	[0,81 - 1,83]
pSB	Level 3	1,25	0,22	0,13	[0,70 - 1,79]
pSB	All	1,24	0,18	0,06	[1,11 - 1,38]
pAN	Level 1	1,43	0,12	0,07	[1,13 - 1,73]
pAN	Level 2	1,37	0,20	0,12	[0,87 - 1,87]
pAN	Level 3	1,47	0,22	0,13	[0,93 - 2,01]
pAN	All	1,42	0,17	0,06	[1,30 - 1,55]
AKR1	-	1,53	0,18	0,10	[0,18 - 0,10]

Supplementary Table S8. *E. coli* growth rate values.
Mean, standard deviation (STD), standard error of the mean(SEM) and 95% confidence intervals are shown for each plasmid level and group. AKR1 does not contain 'Level' plasmids, thus only the group value is reported. Values correspond to three whole independent replicates (N=3).

Plasmid set		DF	SS	MSS	F	PR(>F)
pCA	Level	2.0	0.011597	0.005798	0.203773	0.821067
	Residual	6.0	0.170733	0.028456		
pCO	Level	2.0	0.012275	0.006138	0.272106	0.770694
	Residual	6.0	0.135338	0.022556		
pSB	Level	2.0	0.035281	0.01764	0.480012	0.640651
	Residual	6.0	0.220500	0.03675		
pAN	Level	2.0	0.013763	0.006881	0.201582	0.822754
	Residual	6.0	0.204824	0.034137		

Supplementary Table S9. ANOVA test for E. coli plate fluorometry growth rate values.
A two-way ANOVA comparison was performed among L1, L2 and L3 values for each plasmid kit. There was no statistically significant differences. DF: degrees of freedom; SS: sum of squares; MSS: mean sum of squares; F: F statistic value; PR(>F): p-value of F statistic.

N	Level	Pos.	Name	Description	Notes	Submitter/Source
1	L0	AB	AB_J23101	J23101 promoter (E.coli)	L0>L1 Rx control	Matute & Núñez/[4]
2	L0	AB	AB_R0010 (pLac)	R0010 promoter (MoClo: C1)	AmpR	[1]
3	L0	AC	AC_CEN_ARS_HIS	Yeast centromere (PtPBR)		Vince Bielinski
4	L0	AC	AC_p49202	Pt-p49202		Patrick Brunson
5	L0	AC	AC_pFcpB	Pt-pFcpB		Patrick Brunson
6	L0	AC	AC_pH4	Pt-pH4		Patrick Brunson
7	L0	AC	AC_pNR	Pt-pNR		Patrick Brunson
8	L0	AC	AC_pUBQ10	ubiqutin-10 gene promoter (A. thaliana)		Ariel Cerda [5]
9	L0	AC	AC_pTDH3	pTDH3 promoter	SpectR	Matute & Núñez/[6]
10	L0	AC	AC_pTEF1	pTEF1 promoter	SpectR	Matute & Núñez/[6]
11	L0	AF	AF_Spacer1	L0 universal spacer 1		Bernardo Pollak
12	L0	AF	AF_Spacer2	L0 universal spacer 2		Bernardo Pollak
13	L0	AF	AF_2u	2u ori (yeast)	SpectR; Domestication from p426 (Addgene #43803; DiCarlo et al., 2013).	Matute & Núñez/[7]
14	L0	AF	AF_CEN	CEN ori (yeast)	SpectR	Matute & Núñez/[6]
15	L0	AF	AF_URA3	Encodes URA3	SpectR	Matute & Núñez/[6]
16	L0	BC	BC-B0034m	BBa_B0034m RBS (E. coli)	L0>L1 Rx control	[1]
17	L0	BC	BC_RiboJ54	RiboJ54 F1RBS	AmpR	Matute & Núñez/[4]
18	L0	CD	CD_mRFP1	mRFP1 FP		Bernardo Pollak/[8]
19	L0	CD	CD_Venus	Venus FP		Bernardo Pollak
20	L0	CD	CD_MitoTP	Pt Mitochondrial targeting peptide		Chris Dupont
21	L0	CD	CD_mTagBFP2	mTagBFP2, blue FP	STOP, AmpR. Chemical synthesis from sequence in Subach et al (2011).	Matute & Núñez/[9]
22	L0	CD	CD_mTurquoise2 (yeast)	CDS mTurquoise2 (yeast)	SpectR	Matute & Núñez/[6]
23	L0	CD	CD_ E0030 (YFP)	E0030 (MoClo: E4)	AmpR	[1]
24	L0	CD	CD_mBeRFP	mBeRFP FP	AmpR	Matute & Núñez/[4]
25	L0	CD	CD_sfGFP	sfGFP FP	SpectR	Bernardo Pollak
26	L0	CD	CD_Tur2	mTurquoise2 FP	AmpR	Matute & Núñez/[4]
27	L0	CD	CD_LuxR	C0062 (MoClo: E1)	AmpR	[1]
28	L0	CE	CE_OriTv2	OriT v.2		Vince Bielinski
29	L0	CE	CE_sfGFP	sfGFP FP, green FP	L0>L1 Rx control, STOP	Bernardo Pollak/[10]
30	L0	CE	CE_mRuby3	mRuby3 red FP		Bernardo Pollak/[11]
31	L0	DE	DE_mNeonGreen	mNeonGreen FP		Bernardo Pollak
32	L0	DE	DE_mTurquoise2	mTurquoise2 cyan FP		Bernardo Pollak/ [10]
33	L0	DE	DE_sfGFP	sfGFP FP, green FP		Bernardo Pollak/[10]
34	L0	DE	DE_Venus-ThrHISFLAG	Venus YFP with ThrHisFLAG		Vince Bielinski
35	L0	DE	DE_Venus	Venus YFP		Bernardo Pollak
36	L0	DE	DE_3xStop	3xStop codon - small part		Bernardo Pollak
37	L0	DE	DE_peroxTP	Pt Peroxisomal targetting peptide		Chris Dupont
38	L0	EF	EF_PtBle	ShBle with FcpF promoter		Vince Bielinski
39	L0	EF	EF_B0015	BBa_B0015	L0>L1 Rx control	iGEM
40	L0	EF	EF_t49202	Pt-t49202		Patrick Brunson
41	L0	EF	EF_tFcpB	Pt-tFcpB		Patrick Brunson
42	L0	EF	EF_tH4	Pt-tH4		Patrick Brunson
43	L0	EF	EF_tNR	Pt-tNR		Patrick Brunson
44	L0	EF	EF_tUbq3	Polyubiquitin 3 terminator		plasmid [12]
45	L0	EF	EF_tNos-t35S	nosT-35ST double terminator		Bernardo Pollak [3]
46	L0	EF	EF_t35S	CaMV 35S terminator		Bernardo Pollak/[13]
47	L0	EF	EF_thsp18.2(GB0035)	Hsp18.2 terminator		GB 2.0 kit
48	L0	EF	EF_tADH1	tADH1 terminator	SpectR; Chemical synthesis	Matute & Núñez
49	L0	EF	EF_tCYC1	tCYC1 terminator	SpectR; Domestication from p426 (Addgene #43803; [7]).	Matute & Núñez/[7]
50	L0	*	Venus	Venus FP		Bernardo Pollak/[14]
51	L0	*	mTurquoise2	mTurquoise2 cyan FP		Bernardo Pollak/[15]
52	L0	*	mTagBFP2	mTagBFP2 blue FP		Bernardo Pollak/[9]
53	L0	*	Lti6b-Tag	Lti6b Membrane targetting peptide		Bernardo Pollak/[16]
54	L0	*	N7	N7 Nuclear targetting peptide		Bernardo Pollak/[16]

Supplementary Table S10. L0 part list.

N	Level	Pos.	Name	Description	P1	P2	P3	P4	P5	Rec
1	L1	1	pCAL1-1_PTCv2	Conjugation and prop. Elements	AC_CEN_ARS_HIS	CE_OriTv2	EF_PtBle			pCAo-1
2	L1	2	pCAL1-2_pNR-Venus-PeroxTP-tNR	pNR driven peroxisome Venus	AC_pNR	CD_Venus	DE_peroxTP	EF_tNR		pCAo-2
3	L1	3	pCAL1-3_pH4-MitoTP-mTurquoise2-nH4	pH4 driven mitochondrial mTurquoise2	AC_pH4	CD_MitoTP	DE_mTurquoise2	EF_tH4		pCAo-3
4	L1	4	pCAL1-4_p49202-mTagBFP2-t49202	p49202 driven cytoplasmic mTagBFP2	AC_p49202	CD_mTagBFP2	DE_3xStop	EF_t49202		pCAo-4
5	L1	1	pCAL1-1_pUBQ10-Venus-Lti6b-Ubq3T	pUBQ10 driven membrane Venus	AC_pUBQ10	Venus	Lti6b-Tag	EF_tUbq3		pCAo-1
6	L1	2	pCAL1-2_pUBQ10-mTurquoise2-N7-nosT-35ST	pUBQ10 driven nuclear mTurquoise2	AC_pUBQ10	mTurquoise2	N7	EF_tNos-t35S		pCAo-2
7	L1	3	pCAL1-3_pUBQ10-mRuby3-35ST	pUBQ10 driven cytoplasmic mRuby3	AC_pUBQ10	CE_mRuby3	EF_t35S			pCAo-3
8	L1	4	pCAL1-4_pUBQ10-mTagBFP2-Lti6B - THsp18.2	pUBQ10 driven membrane mTagBFP2	AC_pUBQ10	mTagBFP2	Lti6b-Tag	EF_thsp18.2(GB0035)		pCAo-4
9	L1	1	pSBL1-1_pUBQ10-Venus-Lti6b-Ubq3T	pUBQ10 driven membrane Venus	AC_pUBQ10	Venus	Lti6b-Tag	EF_tUbq3		pSBo-1
10	L1	2	pSBL1-2_pUBQ10-mTurquoise2-N7-nosT-35ST	pUBQ10 driven nuclear mTurquoise2	AC_pUBQ10	mTurquoise2	N7	EF_tNos-t35S		pSBo-2
11	L1	3	pSBL1-3_pUBQ10-mRuby3-35ST	pUBQ10 driven cytoplasmic mRuby3	AC_pUBQ10	CE_mRuby3	EF_t35S			pSBo-3
12	L1	4	pSBL1-4_pUBQ10-mTagBFP2-Lti6B - THsp18.2	pUBQ10 driven membrane mTagBFP2	AC_pUBQ10	mTagBFP2	Lti6b-Tag	EF_thsp18.2(GB0035)		pSBo-4
13	L1	1	pCAL1-1_2u	L1-1 2u ori (yeast)	AF_2u	-	-	-	-	pCAo-1
14	L1	1	pCAL1-1_CEN	L1-1 CEN ori (yeast)	AF_CEN	-	-	-	-	pCAo-1
15	L1	2	pCAL1-2_pTDH3-mTagBFP2-tCYC1	L1-2 pTDH3 encodes mTagBFP2	AC_pTDH3	CD_mTagBFP2	DE_3xStop	EF_tCYC1	-	pCAo-2
16	L1	2	pCAL1-2_pTDH3-mTurq2-tCYC1	L1-2 pTDH3 encodes mTurquoise2	AC_pTDH3	CD_mTurquoise2 (yeast)	DE_3xStop	EF_tCYC2	-	pCAo-2
17	L1	2	pCAL1-2_pTEF1-LuxR-tADH1	L1-2 pTEF1 encodes LuxR	AC_pTEF1	CD_LuxR	DE_3xStop	EF_tADH1	-	pCAo-2
18	L1	4	pCAL1-4_Spacer2	L1-4 Spacer2	AF_Spacer2	-	-	-	-	pCAo-4
19	L1	3	pCAL1-3_URA3	L1-3 URA3	AF_URA3	-	-	-	-	pCAo-3
20	L1	1	pCAL1-1_pLac-54-BeRFP-B0015	L1-1 pLac encodes mBeRFP	AB_R0010	BC_RiboJ54	CD_mBeRFP	DE_3xStop	EF_B0015	pCAo-1
21	L1	2	pCAL1-2_pLac-54-YFP-B0015	L1-2 pLac encodes YFP	AB_R0010	BC_RiboJ54	CD_ E0030 (YFP)	DE_3xStop	EF_B0015	pCAo-2
22	L1	3	pCAL1-3_pLac-54-mTagBFP2-B0015	L1-3 pLac encodes mTagBFP2	AB_R0010	BC_RiboJ54	CD_mTagBFP2	DE_3xStop	EF_B0015	pCAo-3
23	L1	4	pCAL1-4_pLac-54-sfGFP-B0015	L1-4 pLac encodes sfGFP	AB_R0010	BC_RiboJ54	CD_sfGFP	DE_3xStop	EF_B0015	pCAo-4
24	L1	4	pCAL1-4_pLac-54-turq2-B0015	L1-4 pLac encodes mTurquoise2	AB_R0010	BC_RiboJ54	CD_Tur2	DE_3xStop	EF_B0015	pCAo-4
25	L1	1	pSBL1-1_pLac-54-BeRFP-B0015	L1-1 pLac encodes mBeRFP	AB_R0010	BC_RiboJ54	CD_mBeRFP	DE_3xStop	EF_B0015	pSBo-1
26	L1	2	pSBL1-2_pLac-54-YFP-B0015	L1-2 pLac encodes YFP	AB_R0010	BC_RiboJ54	CD_ E0030 (YFP)	DE_3xStop	EF_B0015	pSBo-2
27	L1	3	pSBL1-3_pLac-54-mTagBFP2-B0015	L1-3 pLac encodes mTagBFP2	AB_R0010	BC_RiboJ54	CD_mTagBFP2	DE_3xStop	EF_B0015	pSBo-3
28	L1	4	pSBL1-4_pLac-54-sfGFP-B0015	L1-4 pLac encodes sfGFP	AB_R0010	BC_RiboJ54	CD_sfGFP	DE_3xStop	EF_B0015	pSBo-4
30	L1	4	pSBL1-4_pLac-54-turq2-B0015	L1-4 pLac encodes mTurquoise2	AB_R0010	BC_RiboJ54	CD_Tur2	DE_3xStop	EF_B0015	pSBo-4
31	L1	3	pCAL1-3_Spacer 1	L1-3 Spacer 1	AF_Spacer1	-	-	-	-	pCAo-3
32	L1	2	pCAL1-2_p49202-LuxR-t49202	L1-2 p49202 encodes LuxR	AC_p49202	CD_LuxR	DE_3xStop	EF_t49202	-	pCAo-2
33	L1	2	pCAL1-2_pH4-mTagBFP2-tH4	L1-2 pH4 encodes mTagBFP2	AC_pH4	CD_mTagBFP2	DE_3xStop	EF_tH4	-	pCAo-2
34	L1	1	pL1-1_35SmR3	L1-1_35SmR3	CaMV35S	mRuby3	nosT		-	pOdd-1
35	L1	2	pL1-2_35SmT2	L1-2_35SmT2	CaMV35S	mTurquoise2	N7	nosT	-	pOdd-2
36	L1	3	pL1-3_35SVe	L1-3_35SVe	CaMV35S	Venus	N7	nosT	-	pOdd-3
37	L1	4	pL1-4_35SmR3	L1-4_35SmR3	CaMV35S	mRuby3	nosT		-	pOdd-4
38	L1	4	pCAL1-4_sfGFP	L1 assembly sfGFP test cassette	AB_J23101	BC-B0034m	CE_sfGFP	EF_B0015	-	pCAo-4
39	L1	4	pCOL1-4_sfGFP	L1 assembly sfGFP test cassette	AB_J23101	BC-B0034m	CE_sfGFP	EF_B0015	-	pCOo-4
40	L1	4	pSBL1-4_sfGFP	L1 assembly sfGFP test cassette	AB_J23101	BC-B0034m	CE_sfGFP	EF_B0015	-	pSBo-4
41	L1	4	pANL1-4_sfGFP	L1 assembly sfGFP test cassette	AB_J23101	BC-B0034m	CE_sfGFP	EF_B0015	-	pANo-4

Supplementary Table S11. L1 assembly list.

N	Level	Pos.	Name	Description	P1	P2	P3	P4	Rec
1	L2	1	pCAL2-1_4xFP	L2-1 Fluorescent protein reporters 4x (plant)	pCAL1-1_pUBQ10-Venus-LTi6b	pCAL1-2_pUBQ10-mTurquoise2-N7	pCAL1-3_pUBQ10-mRuby3	pCAL1-4_pUBQ10-mTagBFP2-LTi6B	pCAe-1
2	L2	1	pSBL2-1_4xFP	L2-1 Fluorescent protein reporters 4x (plant)	pSBL1-1_pUBQ10-Venus-LTi6b	pSBL1-2_pUBQ10-mTurquoise2-N7	pSBL1-3_pUBQ10-mRuby3	pSBL1-4_pUBQ10-mTagBFP2-LTi6B	pSBe-1
3	L2	1	pCAL2-1_Yeast-NF	L2-1 pTEF1 encodes LuxR (yeast)	pCAL1-1_CEN	pCAL1-2_pTEF1-LuxR-tADH1	pCAL1-3_URA3	pCAL1-4_Spacer2	pCAe-1
4	L2	1	pCAL2-1_Yeast-mT2	L2-1 Fluorescent reporter mTurquoise2 (yeast)	pCAL1-1_2u	pCAL1-2_pTDH3-mTurq2-tCYC1	pCAL1-3_URA3	pCAL1-4_Spacer2	pCAe-1
5	L2	1	pSBL2-1_Yeast-B	L2-1 Fluorescent reporter mTagBFP2 (yeast)	pCAL1-1_2u	pCAL1-2_pTDH3-mTagBFP2-tCYC1	pCAL1-3_URA3	pCAL1-4_Spacer2	pSBe-1
6	L2	1	pCAL2-1_RYBG	L2-1 Fluorecent protein reporters (E.coli)	pCAL1-1_pLac-54-BeRFP-B0015	pCAL1-2_pLac-54-YFP-B0015	pCAL1-3_pLac-54-mTagBFP2-B0015	pCAL1-4_pLac-54-sfGFP-B0015	pCAe-1
7	L2	1	pCAL2-1_RYBmT2	L2-1 Fluorecent protein reporters (E.coli)	pCAL1-1_pLac-54-BeRFP-B0015	pCAL1-2_pLac-54-YFP-B0015	pCAL1-3_pLac-54-mTagBFP2-B0015	pCAL1-4_pLac-54-turq2-B0015	pCAe-1
9	L2	1	pSBL2-1_RYBG	L2-1 Fluorecent protein reporters (E.coli)	pSBL1-1_pLac-54-BeRFP-B0015	pSBL1-2_pLac-54-YFP-B0015	pSBL1-3_pLac-54-mTagBFP2-B0015	pSBL1-4_pLac-54-sfGFP-B0015	pSBe-1
10	L2	1	pSBL2-1_RYBmT2	L2-1 Fluorecent protein reporters (E.coli)	pSBL1-1_pLac-54-BeRFP-B0015	pSBL1-2_pLac-54-YFP-B0015	pSBL1-3_pLac-54-mTagBFP2-B0015	pSBL1-4_pLac-54-turq2-B0015	pSBe-1
12	L2	1	pSBL2-1_Pt-B	L2-1 Pt Fluorescent reporter mTagBFP2	pCAL1-1_PTCv2	pCAL1-2_pH4-mTagBFP2-tH4	pCAL1-3_Spacer 1	pCAL1-4_Spacer2	pSBe-1
13	L2	1	pCAL2-1_Pt-NF	L2-1 Pt p49202 encodes LuxR	pCAL1-1_PTCv2	pCAL1-2_p49202-LuxR-t49202	pCAL1-3_Spacer 1	pCAL1-4_Spacer2	pCAe-1
14	L2	1	pCAL2-1_FPrep	L2-1 Pt multi-spectral reporter	pCAL1-1_PTCv2	pCAL1-2_pNR-Venus-PeroxTP	pCAL1-3_pH4-MitoTP-mTurquoise2	pCAL1-4_p49202-mTagBFP2	pCAe-1
15	L2	1	pL2-1_all	L2-1 encodes 4 plant FP reporters	pL1-1_35SmR3	pL1-2_35SmT2	pL1-3_35SVe	pL1-4_35SmR3	pEven-1
16	L2	2	pL2-2_all	L2-2 encodes 4 plant FP reporters	pL1-1_35SmR3	pL1-2_35SmT2	pL1-3_35SVe	pL1-4_35SmR3	pEven-2
17	L2	3	pL2-3_all	L2-3 encodes 4 plant FP reporters	pL1-1_35SmR3	pL1-2_35SmT2	pL1-3_35SVe	pL1-4_35SmR3	pEven-3
18	L2	4	pL2-4_all	L2-4 encodes 4 plant FP reporters	pL1-1_35SmR3	pL1-2_35SmT2	pL1-3_35SVe	pL1-4_35SmR3	pEven-4
19	L2	4	pCAL2-4_sfGFP	4 TU construct with sfGFP cassette	pL1-1_35SmR3	pL1-2_35SmT2	pL1-3_35SVe	pCAL1-4_sfGFP	pCAe-4
20	L2	4	pCOL2-4_sfGFP	4 TU construct with sfGFP cassette	pL1-1_35SmR3	pL1-2_35SmT2	pL1-3_35SVe	pCAL1-4_sfGFP	pCOe-4
21	L2	4	pSBL2-4_sfGFP	4 TU construct with sfGFP cassette	pL1-1_35SmR3	pL1-2_35SmT2	pL1-3_35SVe	pCAL1-4_sfGFP	pSBe-4
22	L2	4	pANL2-4_sfGFP	L2 assembly test construct with sfGFP cassette	pL1-1_35SmR3	pL1-2_35SmT2	pL1-3_35SVe	pCAL1-4_sfGFP	pANe-4
23	L3	4	pCAL3-4_sfGFP	16 TU construct with sfGFP cassette	pL2-1_all	pL2-2_all	pL2-3_all	pCAL2-4_sfGFP	pCAo-4
24	L3	4	pCOL3-4_sfGFP	16 TU construct with sfGFP cassette	pL2-1_all	pL2-2_all	pL2-3_all	pCAL2-4_sfGFP	pCOo-4
25	L3	4	pSBL3-4_sfGFP	16 TU construct with sfGFP cassette	pL2-1_all	pL2-2_all	pL2-3_all	pCAL2-4_sfGFP	pSBo-4
26	L3	4	pANL3-4_sfGFP	16 TU construct with sfGFP cassette	pL2-1_all	pL2-2_all	pL2-3_all	pCAL2-4_sfGFP	pANo-4
27	L3	1	pCAL3-1_allx4	16 TU test assembly construct	pL2-1_all	pL2-2_all	pL2-3_all	pL2-4_all	pCAo-1
28	L3	2	pCAL3-2_allx4	16 TU test assembly construct	pL2-1_all	pL2-2_all	pL2-3_all	pL2-4_all	pCAo-2
29	L3	3	pCAL3-3_allx4	16 TU test assembly construct	pL2-1_all	pL2-2_all	pL2-3_all	pL2-4_all	pCAo-3
30	L4	-	L4-4_sfGFP	64 TU 4 piece assembly	pCAL3-1_allx4	pCAL3-2_allx4	pCAL3-3_allx4	pCAL3-4_sfGFP	-

Supplementary Table S12. L2, L3 and L4 assembly list

Plate fluorometry data analysis

Dynamical measurements of bacterial growth (OD600 absorbance) and fluorescence obtained by plate reader fluorometry were analysed with python routines included in Jupyter notebooks described below.
load-data.ipynb : was used to load and format the data from the plate reader to the experimental database.
data-treatment.ipynb: was used to subtract absorbance and fluorescence background values.
data-analysis-uLoop.ipynb: was used to organise data, apply models to obtain relevant parameters, perform the statistics and make the plots.
Please read the comments in the code for further details [17].
Signal readings (I_m) were cleaned by subtracting the corresponding background (I_b) values in each case as shown in equation 1.

$$I_i(t) = I_m(t) + I_b(t). \tag{1}$$

Next, cross measurements of red and green fluorescence from GFP and RFP control cultures, respectively, were performed to determine any bleed-through signal. Red and green fluorescence signals were measured from cell cultures containing pCAL1-4-sfGFP plasmid and from AKR1 cell cultures, respectively. Empirical functions were fitted to each data set as shown in Supplementary Figures S**??** and Figures S**??**, which relate bleed-through fluorescent signal with growth (OD600). These functions were applied to each data series to subtract the proper background signals (Figures S**??** and Figures S**??**). Relevant parameters related to fluorescent expression were extracted from pre-treated data. As sfGFP and RFP genes were under constitutive expression, they were expected to display a constant fluorescent ratio. In accordance to this, sfGFP signal followed roughly a linear relation with respect to mRFP1 signal (Supplementary Figures S6-S10). Next, a linear fit was performed to characterise each data set as the expression ratio obtained from the slope of these linear functions (Supplementary Figures S6-S10). Mean ratio values were computed by vector set and assembly level over the 3 whole replicates (N=3 and 6 technical replicates of each day)(Figure 4A and Supplementary table S1). To measure the variability of expression for each vector set, the coefficient of variation (standard deviation divided by the mean reported as a percentage) was computed for each group (Supplementary table S7).

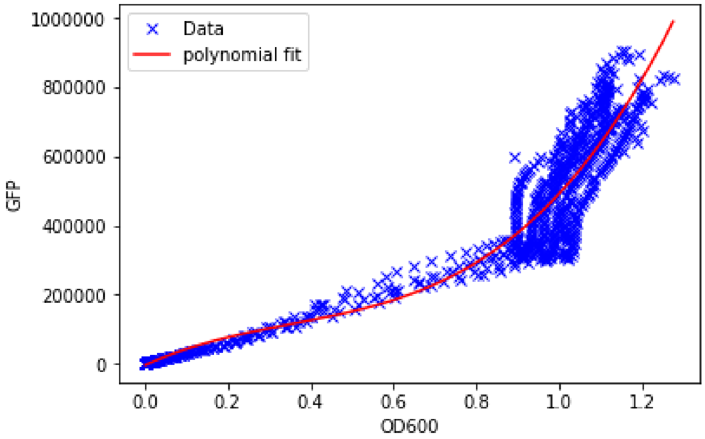

Supplementary Figure S9. AKR1 green bleed-trough estimation.
An empirical function was fitted to GFP (ex: 485 em: 516) vs OD600 from AKR1 control cultures (N = 3). Blue crosses correspond to each data point and the red line represents the fitted polynomial function.

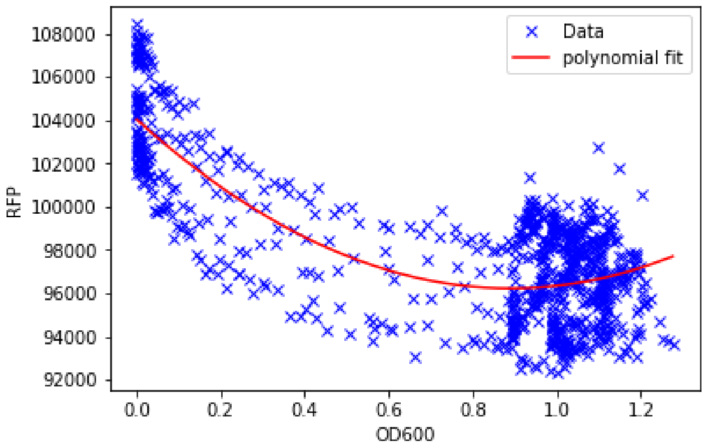

Supplementary Figure S10. Red fluorescence bleed-trough estimation.
An empirical function was fitted to RFP (ex: 585 em: 620) vs OD600 from AKR1 control cultures (N = 3). Blue crosses correspond to each data point and the red line represents the fitted polynomial function.

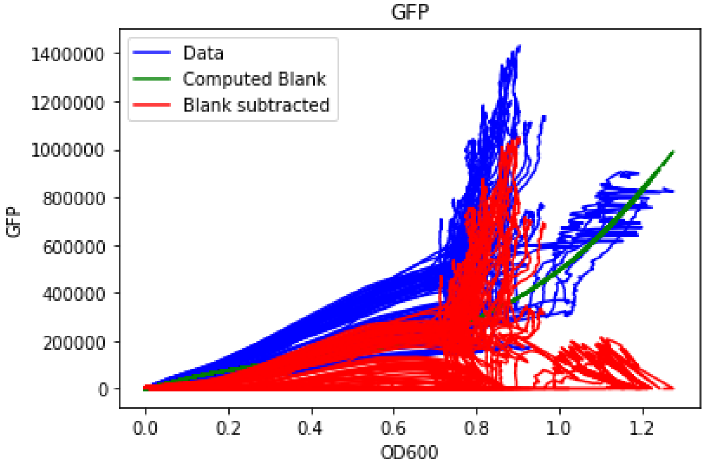

Supplementary Figure S11. GFP background subtraction.
A fitted function was used to remove the background from GFP measurements for each data set. Blue lines correspond to raw GFP values, the green line corresponds to the empirical background function and red lines represent background-subtracted data.

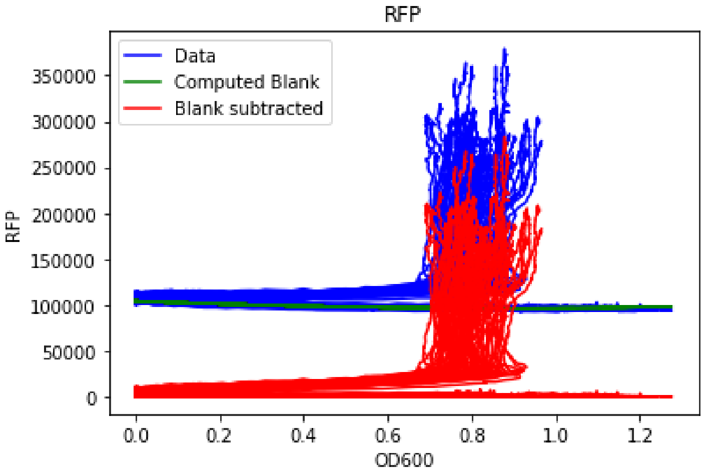

Supplementary Figure S12. RFP background subtraction.
A fitted function was used to remove the background from RFP measurements for each data set. Blue lines correspond to raw RFP values, the green line represents the empirical background function and red lines correspond to background-subtracted data.

Growth rate measurements

First, blank wells containing M9-Glycerol medium only were measured to obtain absorbance background values. As the OD600 blank values were small and approximately constant, a single mean was taken and used as a background value (OD600 blank value = 0.081 units) and later subtracted to all the other well values. Next, the effect of different plasmid kits and levels on cell growth was analysed by fitting a Gompertz model [18] over OD600 absorbance data for each well, according to equation 2:

$$ln\left[\frac{A}{A_0}\right] = k \cdot exp\left\{ - exp\left[\frac{\mu_{max} \cdot e}{k} \cdot (\lambda - t) + 1\right]\right\} \tag{2}$$

The maximum growth rate parameter (μ_{max}) was obtained for each data series. They were grouped and analysed by level and vector kit as shown in Figure 4C and supplementary Table S3.

References

[1] Sonya V. Iverson, Traci L. Haddock, Jacob Beal, and Douglas M. Densmore. Cidar moclo: Improved moclo assembly standard and new e. coli part library enable rapid combinatorial design for synthetic and traditional biology. ACS Synthetic Biology, 5(1):99–103, 01 2016.

[2] Nicola J. Patron, Diego Orzaez, Sylvestre Marillonnet, Heribert Warzecha, Colette Matthewman, Mark Youles, Oleg Raitskin, Aymeric Leveau, Gemma Farré, Christian Rogers, Alison Smith, Julian Hibberd, Alex A. R. Webb, James Locke, Sebastian Schornack, Jim Ajioka, David C. Baulcombe, Cyril Zipfel, Sophien Kamoun, Jonathan D. G. Jones, Hannah Kuhn, Silke Robatzek, H. Peter Van Esse, Dale Sanders, Giles Oldroyd, Cathie Martin, Rob Field, Sarah O'Connor, Samantha Fox, Brande Wulff, Ben Miller, Andy Breakspear, Guru Radhakrishnan, Pierre-Marc Delaux, Dominique Loqué, Antonio Granell, Alain Tissier, Patrick Shih, Thomas P. Brutnell, W. Paul Quick, Heiko Rischer, Paul D. Fraser, Asaph Aharoni, Christine Raines, Paul F. South, Jean-Michel Ané, Björn R. Hamberger, Jane Langdale, Jens Stougaard, Harro Bouwmeester, Michael Udvardi, James A. H. Murray, Vardis Ntoukakis, Patrick Schäfer, Katherine Denby, Keith J. Edwards, Anne Osbourn, and Jim Haseloff. Standards for plant synthetic biology: a common syntax for exchange of dna parts. New Phytologist, 208(1):13–19, 2019/08/10 2015.

[3] Bernardo Pollak, Ariel Cerda, Mihails Delmans, Simón Álamos, Tomás Moyano, Anthony West, Rodrigo A. Gutiérrez, Nicola J. Patron, Fernán Federici, and Jim Haseloff. Loop assembly: a simple and open system for recursive fabrication of dna circuits. New Phytologist, 222(1):628–640, 2019/07/19 2019.

[4] Isaac Nuñez, Tamara Matute, Roberto Herrera, Juan Keymer, Timothy Marzullo, Timothy Rudge, and Fernán Federici. Low cost and open source multi-fluorescence imaging system for teaching and research in biology and bioengineering. PLoS One, 12(11):e0187163, 2017.

[5] Christopher Grefen, Naomi Donald, Kenji Hashimoto, Jörg Kudla, Karin Schumacher, and Michael R. Blatt. A ubiquitin-10 promoter-based vector set for fluorescent protein tagging facilitates temporal stability and native protein distribution in transient and stable expression studies. The Plant Journal, 64(2):355–365, 2019/07/19 2010.

[6] Michael E. Lee, William C. DeLoache, Bernardo Cervantes, and John E. Dueber. A highly characterized yeast toolkit for modular, multipart assembly. ACS Synthetic Biology, 4(9):975–986, 09 2015.

[7] James E. DiCarlo, Julie E. Norville, Prashant Mali, Xavier Rios, John Aach, and George M. Church. Genome engineering in saccharomyces cerevisiae using crispr-cas systems. Nucleic Acids Research, 41(7):4336–4343, 7/19/2019 2013.

[8] Fernán Federici, Lionel Dupuy, Laurent Laplaze, Marcus Heisler, and Jim Haseloff. Integrated genetic and computation methods for in planta cytometry. Nature Methods, 9:483 EP –, 04 2012.

[9] Oksana M. Subach, Paula J. Cranfill, Michael W. Davidson, and Vladislav V. Verkhusha. An enhanced monomeric blue fluorescent protein with the high chemical stability of the chromophore. PLOS ONE, 6(12):e28674–, 12 2011.

[10] Timothy J. Rudge, Fernán Federici, Paul J. Steiner, Anton Kan, and Jim Haseloff. Cell polarity-driven instability generates self-organized, fractal patterning of cell layers. ACS Synthetic Biology, 2(12):705–714, 12 2013.

[11] Bryce T. Bajar, Emily S. Wang, Amy J. Lam, Bongjae B. Kim, Conor L. Jacobs, Elizabeth S. Howe, Michael W. Davidson, Michael Z. Lin, and Jun Chu. Improving brightness and photostability of green and red fluorescent proteins for live cell imaging and fret reporting. Scientific Reports, 6:20889 EP –, 02 2016.

[12] Smita Kurup, John Runions, Uwe Köhler, Laurent Laplaze, Sarah Hodge, and Jim Haseloff. Marking cell lineages in living tissues. The Plant Journal, 42(3):444–453, 2019/07/19 2005.

[13] Maciej Pietrzak, Raymond D. Shillito, Thomas Hohn, and Ingo Potrykus. Expression in plants of two bacterial antibiotic resistance genes after protoplast transformation with a new plant expression vector. Nucleic Acids Research, 14(14):5857–5868, 7/19/2019 1986.

[14] Takeharu Nagai, Keiji Ibata, Eun Sun Park, Mie Kubota, Katsuhiko Mikoshiba, and Atsushi Miyawaki. A variant of yellow fluorescent protein with fast and efficient maturation for cell-biological applications. Nature Biotechnology, 20(1):87–90, 2002.

[15] Joachim Goedhart, Laura van Weeren, Mark A Hink, Norbert O E Vischer, Kees Jalink, and Theodorus W J Gadella Jr. Bright cyan fluorescent protein variants identified by fluorescence lifetime screening. Nature Methods, 7:137 EP –, 01 2010.

[16] Sean R. Cutler, David W. Ehrhardt, Joel S. Griffitts, and Chris R. Somerville. Random gfp-cdna fusions enable visualization of subcellular structures in cells of arabidopsis at a high frequency. Proceedings of the National Academy of Sciences, 97(7):3718, 03 2000.

[17] Isaac Nuñez. Github repository for jupyter notebooks. https://github.com/Prosimio/Plate_reader_analysis, 2019. [Online; accessed 13-July-2019].

[18] M H Zwietering, I Jongenburger, F M Rombouts, and K van 't Riet. Modeling of the bacterial growth curve. Applied and environmental microbiology, 56(6):1875–1881, 06 1990.
